# Processing bimodal stimuli: integrality/separability of color and orientation

**DOI:** 10.3389/fpsyg.2013.00759

**Published:** 2013-10-17

**Authors:** David L. Bimler, Chingis A. Izmailov, Galina V. Paramei

**Affiliations:** ^1^School of Arts, Development and Health Education, Massey UniversityPalmerston North, New Zealand; ^2^Department of Psychophysiology, Faculty of Psychology, Moscow Lomonosov State UniversityMoscow, Russia; ^3^Department of Psychology, Liverpool Hope UniversityLiverpool, UK

**Keywords:** color, orientation, bimodal stimuli, feature integration, multidimensional scaling, Minkowski metric, integral dimensions, separable dimensions

## Abstract

We examined how two distinct stimulus features, orientation and color, interact as contributions to global stimulus dissimilarity. Five subjects rated dissimilarity between pairs of bars (*N* = 30) varying in color (four cardinal hues, plus white) and orientation (six angles at 30° intervals). An exploratory analysis with individual-differences multidimensional scaling (MDS) resulted in a 5D solution, with two dimensions required to accommodate the circular sequence of the angular attribute, and red-green, blue-yellow and achromatic axes for the color attribute. Weights of the orientation subspace relative to the color subspace varied among the subjects, from a 0.32:0.61 ratio to 0.53:0.44, emphasis shifting between color and orientation. In addition to Euclidean metric, we modeled the interaction of color and orientation using Minkowski power metrics across a range of Minkowski exponents *p*, including the city-block (*p* = 1), Euclidean (*p* = 2) and Dominance metric (*p* → ∞) as special cases. For averaged data, *p* ~ 1.3 provided the best fit, i.e., intermediate between separable and integral features. For individual subjects, however, the metric exponent varied significantly from *p* = 0.7 to *p* = 3.1, indicating a subject-specific rule for combining color and orientation, as in Tversky and Gati's variable-weights model. No relationship was apparent between dimensional weights and individual *p* exponents. Factors affecting dimensional integrality are discussed, including possible underlying neural mechanisms where the interaction of the low-level vision attributes orientation and color might shift between uncorrelated (*p* = 1) or correlated (*p* ≥ 2) forms.

## Introduction

Researchers in visual perception frequently ask observers whether two stimuli are different, or how different they are. Ecologically-valid stimuli can vary along more than one attribute, or in more than one visual sub-modality: that is, their description requires more than one dimension. The total inter-stimulus dissimilarity is then an aggregate of differences across multiple attributes, and the research question becomes one of how these differences interact.

A research tradition beginning with Attneave (1950) has focused on the special case of “integral” dimensions, where the attributes on which the stimuli are parameterized can be replaced with oblique linear combinations, intrinsically as good as the original parameters, because the inter-stimulus dissimilarities remain the same. The classic examples of integral dimensions are lightness and saturation in color space (e.g., Hyman and Well, 1967; Ashby and Townsend, 1986; Burns and Shepp, 1988). In Garner's words (1974, 199), “[p]sychologically, if dimensions are integral, they are not really perceived as dimensions at all. Dimensions exist for the experimenter [… ] but these are constructs [… ] and do not reflect the immediate perceptual experience of the subject in such experiments ….” That is, integral dimensions form a seamless Gestalt.

A second possibility is that the dimensions do not interact, with dissimilarity perceived as simply a linear summation of the absolute differences on each of the dimensions in isolation. Such non-interacting attributes have been dubbed “separable” or “analyzable” (Attneave, 1950; Shepard, 1964).

The integral/separable distinction is of interest for visual psychophysics because feature interaction is ubiquitous by nature and not restricted to explicit judgments of dissimilarity, but bears upon other tasks, such as stimulus classification (Garner and Felfoldy, 1970; Ashby, 1988); classification errors (Attneave, 1950; Shepard and Chang, 1963); visual search (Treisman and Gormican, 1988; Koene and Zhaoping, 2007). The implications for perceptual mechanisms will be discussed below. However, integral and analyzable dimensions are not the only alternatives. Numerous models of feature integration can be subsumed within a family of Minkowski metrics or “*L*_*p*_ norms,” characterized by a parameter *p*, the Minkowski exponent (e.g., Shepard, 1987). Writing Δ *D*_*n*_ for the difference between two stimuli along the *n*-th dimension:

(1) $$\text{Dissimilarity}={\left[\sum_{n}\Delta D_{n}{}^{p}\right]}^{1/p}$$

Separable dimensions correspond to *p* = 1, the city-block metric (Householder and Landahl, 1945). Higher values define perceptual models where some level of integration, suppression, or competition occurs among the attributes. Integral dimensions are formally described by *p* = 2, the familiar Euclidean metric (the *L*_2_ norm). Here Equation 1 reduces to the Pythagorean formula for distance.

In the limiting case of *p* → ∞, dissimilarity is dictated by the *maximum* of the differences along the separate dimensions or attributes; that is, a dimension is suppressed and does not contribute to the dissimilarity if a larger difference exists along another dimension to dominate it. This has been dubbed the “supremum” or “dominance” metric (e.g., Hyman and Well, 1967). Note that although integer values of *p* receive most attention, the Minkowski framework remains valid for fractional values.

Early examinations of the dissimilarities from composite changes used simple stimuli (circle-spoke structures) confined to a single location in the visual field (Shepard, 1964; Garner and Felfoldy, 1970). Judgments of more elaborated attribute combinations were subsequently considered. Griffin and Sepehri (2002) used pairwise comparisons of simple stimuli varying in texture and color, concluding that the interaction follows a Minkowski metric (though they did not specify the value of *p*). Izmailov and Edrenkin (2010) elicited dissimilarity ratings among bar stimuli combining orientation and luminance and found *p* in the range 1.8–2.0.

In contrast to the above simple-stimulus studies, To et al. (2008, 2010) collected dissimilarity judgments for pairs of natural visual scenes that had been subjected to one or two ecologically-realistic manipulations (color, location, size, and/or blur). The image manipulations were typically distributed across the entire scene and could not be parameterized by or reduced to a change in a single localized “visual primitive.” The authors' analysis led to the conclusion that dissimilarities between images separated by two manipulations were most concordant with those between single-manipulation pairs if the latter interacted according to a quasi-Dominance metric, with *p* = 2.84 (To et al., 2008) or *p* = 2.48 (To et al., 2010).

Apart from its psychophysical meaning, the *p* exponent may reflect the mechanisms of cortical processing accessed by a given task or index of dissimilarity. To et al. (2008, 2011) proposed that a large *p* can be expected when the variations along the dimensions are strongly correlated, exhibiting a high level of redundancy, so that a stimulus difference along one attribute is normally accompanied by a comparable difference on the others. In informational terms, such differences can be encoded efficiently if the neural nexus at which they converge allows the larger of the signals to dominate others that add little or no further information about dissimilarity.

Conversely, the additive combination expressed as the city-block metric is the most efficient way of encoding a combination of difference signals from attributes where the values are empirically uncorrelated. If visual primitives such as color, size, or orientation are processed independently and in parallel, then the dissimilarity when two such features vary might be a linear combination of the Δ*D*_*n*_ in isolation, i.e., *p* near 1. Intermediate degrees of correlation require intermediate levels of non-linearity: competition among the attributes implies the kind of non-linear combinations characterized by *p* > 1 (Zhaoping and Snowden, 2006).

The present study further explores the interaction of visual attributes in bimodal stimuli and extends Izmailov and Edrenkin's (2010) path of research, with bar stimuli varying in color (rather than luminance) in addition to orientation. To determine *p* for this situation, we obtained the dissimilarity for each difference in orientation independent of color and each difference in color independent of orientation, and used these to predict the dissimilarity between pairs differing in both color *and* orientation, while varying *p* in the Minkowski metric (cf. Shepard and Cermak, 1973; To et al., 2008).

This approach postulates that color and orientation are separable (in the mathematical sense), i.e., that the difference between each same-color pair (differing only in orientation) is constant whatever the color, and conversely for each same-orientation pair. To test this postulate we applied multidimensional scaling (MDS) to the data. Unlike the process discussed so far, MDS begins with a matrix of inter-stimulus dissimilarities or “map distances” and reconstructs “map coordinates”: *empirical* dimensional descriptions of the subjects' mental/perceptual representations of the stimuli. We ask whether a geometrical representation is adequate, treating it as Euclidean in nature (*p* = 2). It is tempting to seek the Minkowski metric for a given set of data by repeating MDS analysis for different *p*, and choosing the value that minimizes the mismatch between data and reconstructed distances. However, this strategy is known to be deceptive (Arnold, 1971; Shepard, 1974).

## Materials and methods

### Participants

Five participants (four females), aged 20–27 years old, were normal trichromats with normal or corrected-to-normal vision. They were all undergraduate Psychology students, familiar with the scaling procedure but naïve to the specific research area.

### Stimuli

**Figure 1:**
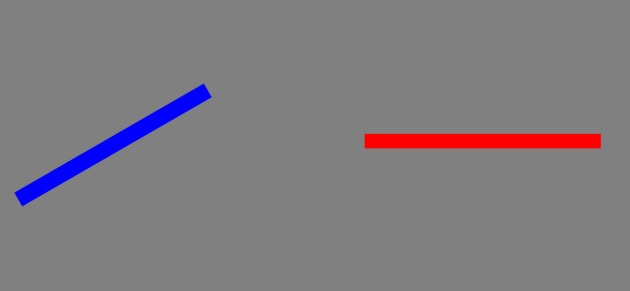
**Example of two stimuli presented for dissimilarity judgment**.

Stimuli were colored bars of different orientation presented on a CRT screen at 12 cd/m^2^ against a darker (2 cd/m^2^) gray background (as illustrated in Figure 1). At the viewing distance of 100 cm, each bar subtended an angle of 8.6° lengthwise and 0.6° widthwise. The bars were presented in pairs, to the right and left from the central nominal fixation point; their *centers* were separated by 10.8°, the same for all pairs—that is, the bars can be imagined as rotating around these centers of gravity to generate the different orientations. Observation was binocular, without head fixation, in an otherwise-unlit room.

The bars took on six orientations, varying in 30° steps from the horizontal, and five different colors: red, yellow, green, blue, and white (Table 1). Thus, the two variables created 30 different bars. For convenience, these are labeled below as S^*a*^_*m*_, where the subscript *m* identifies the orientation and the superscript index *a* identifies the color.

**Table 1:** **CIE 1931 coordinates of the stimulus colors**.

**Color**	***x***	***y***	***L* (cd/m^2^)**
Green	0.900	0.600	12
Blue	0.170	0.145	12
White	0.355	0.355	12
Yellow	0.405	0.510	12
Red	0.620	0.340	12
Dark gray (background)	0.355	0.355	2

### Procedure

Subjects were instructed to rate the total dissimilarity of each pair of bars on a scale of 1 (least) to 9 (most). No particular pair was provided to subjects as an example of the maximum value. Each pair of bars was shown twice to each subject, once in the form “*i:j*” and once as the mirror-image “*j:i*,” providing (30 × 29 =) 870 pairs. These were presented in the course of three sessions for each subject. Each pair was presented for 1.5 s followed by a 0.5 s interval, during which the subject entered the rating using corresponding keys of a computer keyboard. The response was not recorded if it exceeded this interval, though for each individual subject the number of missing inputs was just one or two.

The square matrix of pairwise differences obtained from each participant consists of an upper and lower triangular half-matrix containing ***i:j*** and ***j:i*** pairs. The Pearson correlation coefficients *r* between these two values for each participant are shown in the diagonal elements of Table 2 and indicate good intra-subject replicability. Inter-subject replicability, shown in the off-diagonal elements of Table 2, was fair. Here the two judgments from each subject for a given stimulus pair were averaged and compared with the mean from each other subject.

**Table 2:** **Pearson correlation coefficients between individual subjects' dissimilarity matrices and (on the diagonal) between individuals' *ij* and *ji* half-matrices**.

**Ss**	**1**	**2**	**3**	**4**	**5**
1	0.86	0.76	0.55	0.61	0.54
2		0.67	0.49	0.48	0.39
3			0.48	0.37	0.43
4				0.66	0.58
5					0.55

**Figure 2:**
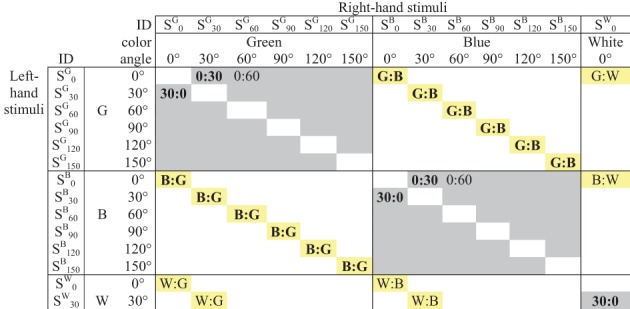
**Pairwise comparisons of stimuli created from two attributes (upper left corner of matrix only).** Gray cells indicate *orientation-only* pairs S^*a*^_*m*_: S^*a*^_*n*_; yellow cells indicate *color-only* pairs S^*a*^_*m*_: S^*b*^_*m*_. The Figure shows the 12 pairs averaged for the estimated Green:Blue dissimilarity; five of the 10 pairs averaged to estimate the 30°:0° dissimilarity; etc.

The 870 stimulus pairs can be classified into three classes, as shown in Figure 2:

1. 150 *orientation-only* pairs of the form (S^*a*^_*m*_: S^*a*^_*n*_), in which the bars differ in orientation but are both the same color, “*a*,” out of five possibilities;
2. 120 *color-only* pairs of the form (S^*a*^_*m*_: S^*b*^_*m*_), in which the bars differ in color but are both orientation “*m*,” out of six possibilities;
3. 600 *bimodal* pairs (S^*a*^_*m*_: S^*b*^_*n*_), differing in both orientation and color (for brevity we use “bimodal” in a broader sense than usual since sub-modalities of vision are involved rather than separate sensory modalities).

## Results

Each subject's dissimilarity judgments were analyzed in combination with other subjects, and then in isolation. The mean dissimilarity ratings for each subject, across all 870 pairs, were 5.83, 5.76, 5.84, 5.25, and 4.66. We examine the individual distributions of ratings below (Figure 7), noting for now that all subjects used the full range from 1 to 9. That is, all five subjects used the response scale in much the same manner.

### Multidimensional scaling (euclidean metric)

Analysis began with MDS, in which a Euclidean geometrical model is used to account for the data, representing each stimulus as a point in a low-dimensional space. In an iterative process, the locations of the 30 points are adjusted so that the distances among them reflect the dissimilarities among the corresponding stimuli as accurately as possible. Any mismatch between the data and reconstructed distances in a solution is measured by *stress*_1_, an index of badness-of-fit, which is progressively minimized by the MDS process (Kruskal, 1964). The dimensions of the solution can be interpreted as the variables that underlie the visual domain in question.

As noted above, we work with the assumption of Euclidean geometry, i.e., *p* = 2, secure in the knowledge that departures from this approximation have little effect on MDS solutions (Arabie, 1991). Attempting to accommodate the averaged data within 3D, 4D, 5D, and 6D models resulted in *stress*_1_ values of 0.217, 0.167, 0.129, and 0.112, respectively. Standard rules for interpreting *stress*_1_ (Kruskal, 1964) show the three-dimensional solution to be inadequate. Here we focus on the 5D solution, ignoring the 6D version which provides only a small improvement in goodness-of-fit.

To rotate the optimized solution to non-arbitrary dimensions, we applied the “weighted Euclidean” or INDSCAL framework of individual variation (Wish and Carroll, 1974). This framework allows for the possibility that subjects vary in the relative salience or weight they place on one dimension or another: that is, an inter-stimulus difference along a given dimension may contribute more to perceived dissimilarity for one subject than another. Specifically, the model includes dimensional-weight parameters *w*_*qd*_ (where the index *q* designates a subject, while *d* labels the dimensions), and finds their optimal values. If the coordinates of the *i*-th and *j*-th items in the model are written *x*_*id*_ and *x*_*jd*_ respectively, the parameters *w*_*qd*_ modulate the perceived inter-item distances for that subject:

(2) $$\text{distance}{\left(i,j\right)}^{2}=\sum_{d}w_{qd}^{2}{\left(x_{id}-x_{jd}\right)}^{2}$$

This weighting is equivalent to systemically altering the inter-point distances by stretching or compressing the consensus model along its dimensions for a better fit to each subject's data (which are kept separate in this analysis). The outcome is that the dimensions of the final solution (which would otherwise be arbitrary) correspond to modes of inter-subject variation within the data. To test whether noise alone could account for any differences in the subject-specific weight parameters, the *w*_*qd*_ were replicated by repeating the INDSCAL analysis with each subject's *i:j* and *j:i* matrices treated separately.

### Orientation subspace

**Figure 3:**
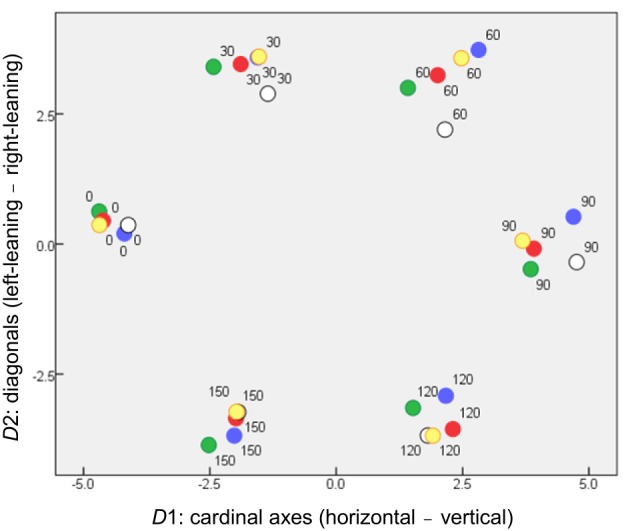
**Locations of stimuli in the orientation subspace of MDS solution (coordinates on *D*1 and *D*2), where symbols are coded according to stimulus color**.

Figure 3 is a scatterplot in which the stimuli are located by their coordinates on the first two (rotated) dimensions, *D*1 and *D*2. These clearly accommodate the orientation parameter. Two dimensions are required rather than one because of that parameter's cyclic nature (for these symmetrical stimuli, θ + 180° is equivalent to θ ), to give the parameter room to loop back on itself. This outcome is in accord with previous results when stimulus pairs of bars varied in orientation alone (Indow, 1988; Izmailov et al., 2004) or in orientation and luminance (Izmailov and Edrenkin, 2010).

Even so, the dimensions have separate physical meanings. *D*1 serves to separate horizontal from vertical stimuli, providing the dissimilarity between what have been called “the cardinal axes of the visual coordinate system” (Orban et al., 1984). *D*2 separates bars inclined right vs. left from the vertical direction. *D*1 disperses the stimuli more than *D*2, accounting for more variance in the MDS solution (27.7% compared to 23.1%). This is consistent with the “oblique effect” (cf. Orban et al., 1984), whereby spatial vision exhibits orientation anisotropy, so that two bars at right angles seem more dissimilar if they align with the cardinal axes than if they are diagonals.

We note also that the dissimilarities among orientations obtained by Izmailov et al. (2004) could best be explained by separate cardinal-axis and diagonal-axis contributions, combining in a Minkowski metric with *p* ~1.75.

### Color subspace

**Figure 4:**
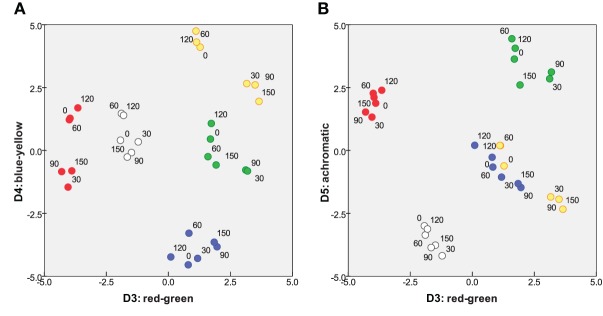
**Two projections of color subspace of 5D MDS solution.** Coordinates of stimuli on **(A)** *D*3 and *D*4, red-green and blue-yellow; and **(B)** *D*3 and *D*5 “achromatic” dimension.

The remaining three dimensions capture the dissimilarities from differences in color. Figure 4 projects the solution onto its 3rd vs. 4th dimensions and 3rd vs. 5th dimensions. In each panel, other dimensions (including *D*1 and *D*2) are orthogonal to the plane of the page. *D*3 and *D*4 can immediately be identified as “red-green” and “blue-yellow” opponent perceptual systems, respectively. Further, it appeared that a white and any chromatic bar are perceived as more dissimilar than the isoluminant plane can accommodate, requiring *D*5, an “achromatic” distinction, to capture this additional dissimilarity.

The crucial aspect of Figures 3, 4 is that subjects treat orientation and color as separate, decoupled attributes in their mental/perceptual models, with each attribute confined to its own subspace, orthogonal to the other subspace. That is, a given pair of orientations are perceived as equally dissimilar if the bars are (for instance) both blue or both yellow. Conversely, dissimilarities within the color subspace are the same whether two colors are presented as a pair of 30° bars or a pair of any other angle. This lack of coupling is a pre-requisite for applying Equation 1 in subsequent analysis.

### Relative salience of the orientation and color subspaces

This rotated 5D solution provides a quantitative measure of the relative importance of the inter-color and inter-orientation differences for the subjects. Specifically, the combined axes of the “orientation subspace” (Figure 3) disperse the items marginally more than the combined axes of the “color subspace” (Figure 4), respectively accounting for 50.2 and 49.8% of variance within the MDS solution. Note that this is a combined outcome, with wide variations in the relative importance of orientation to individual subjects.

### Individual differences in dimension weights

**Figure 5:**
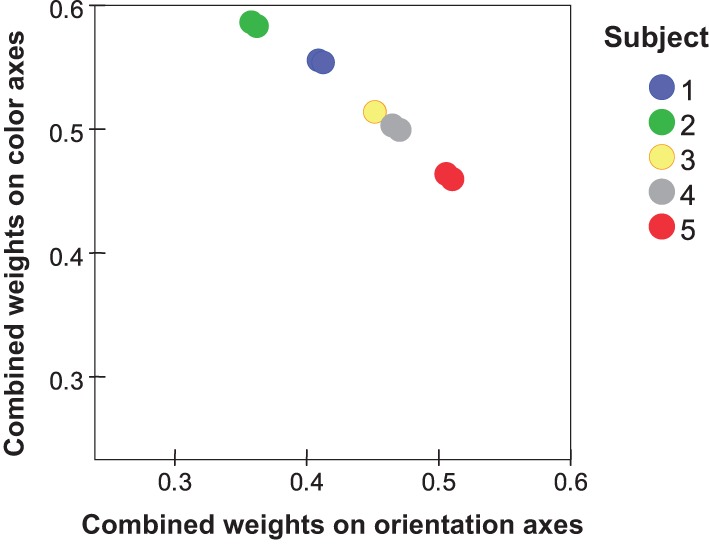
**Individual values from MDS (two values per subject) for the combined weights of orientation dimensions (*w*^2^_*q*1_ + *w*^2^_*q*2_) plotted against the combined weights of color dimensions (*w*^2^_*q*3_ + *w*^2^_*q*4_ + *w*^2^_*q*5_)**.

Table 3 shows dimension weights from the individual-differences MDS analysis. In the orientation subspace, subjects differed in the weights they placed on *D*1 (cardinal axes) relative to those for *D*2 (diagonals). Greater variations appeared in the color subspace, particularly in the weight of *D*3, red-green dimension, relative to the blue-yellow (*D*4) and achromatic (*D*5) dimensions. Notably, the combined weight of the orientation subspace relative to the color subspace (Table 3) again showed individual variations (Figure 5). These differences are replicated between *i:j* and *j:i* ratings. Subject #5, for instance, places relatively greater weight on orientation differences while Subjects #1 and #2 place more weight on color differences. Here the combined weights of the orientation and color subspaces are *w*_*q*O_ = (*w*^2^_*q*1_ + *w*^2^_*q*2_)^0.5^ and *w*_*qC*_ = (*w*^2^_*q*3_ + *w*^2^_*q*4_ + *w*^2^_*q*5_)^0.5^, respectively.

**Table 3:** **Parameters of individual and mean data from the MDS and Minkowski-metric analyses**.

**Subject**	***Stress*_1_ (in 5D)**	***D*1 card**	***D*2 diag**	***D*3 R-G**	***D*4 B-Y**	***D*5 Achr.**	**Subspace weights: orientation/color**	**Mean Δ_mn_ (orientation)**	**Mean Δ^ab^ (color)**	**Optimal Minkowski *p***
1	0.10	0.31	0.29	0.31	0.34	0.30	0.41/0.56	2.60	5.00	1.3
2	0.07	0.29	0.22	0.34	0.34	0.34	0.32/0.61	3.00	5.57	1.8
3	0.15	0.34	0.30	0.31	0.31	0.29	0.46/0.51	4.22	5.65	3.1
4	0.12	0.35	0.32	0.30	0.28	0.29	0.47/0.50	3.64	1.40	0.7
5	0.09	0.38	0.35	0.27	0.26	0.27	0.53/0.44	3.67	2.10	1.1
Mean	0.13							3.43	3.94	1.3

5D solution for individual-differences MDS includes weights w_q^1^_ … w_q^5^_ on the five dimensions (two orientation, three chromatic); total weight (w^2^_q1_ + w^2^_q2_)^0.5^ of orientation subspace; total weight (w^2^_q3_ + w^2^_q4_ + w^2^_q5_)^0.5^ of color subspace.

### Dissimilarity judgments for color and for orientation

**Figure 6:**
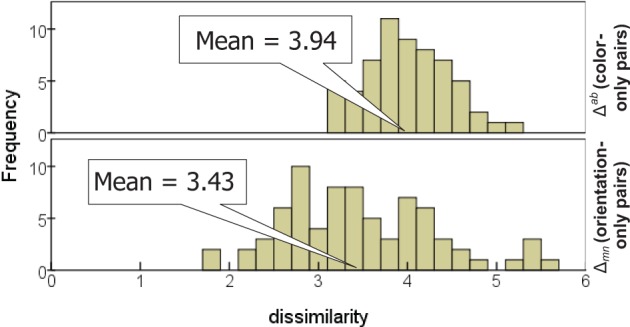
**Distribution of dissimilarities for stimulus pairs differing in color only (top) and orientation only (bottom)**.

Averaged across the subjects, the dissimilarities for orientation and for color are comparable in magnitude (Figure 6), with the mean rating across *color-only* pairs (3.94 ± 0.47) slightly greater than the mean across *orientation-only* pairs (3.43 ± 0.85). Recall that *orientation-only* pairs outnumber *color-only* pairs (150 vs. 120), and contribute more to variance; thus this is consistent with the earlier observation that the color subspace disperses the items slightly less than the orientation subspace.

Table 3 indicates substantial inter-individual variation, however, with Subject #2 rating *color-only* pairs twice as dissimilar as *orientation-only* pairs, while for other subjects they are only half as dissimilar (also evident in the dimensional weights).

We mention this situation of similar magnitude for *color-only* and *orientation-only* dissimilarities because it provides greatest sensitivity to *p* in the comparison between predicted and actual dissimilarities (cf. To et al., 2008, 2010). If *p* > 1 (i.e., if there is some degree of non-linear competition between the single-attribute differences), and if either attribute is generally smaller than the other, it contributes disproportionately less to the combined dissimilarity.

**Figure 7:**
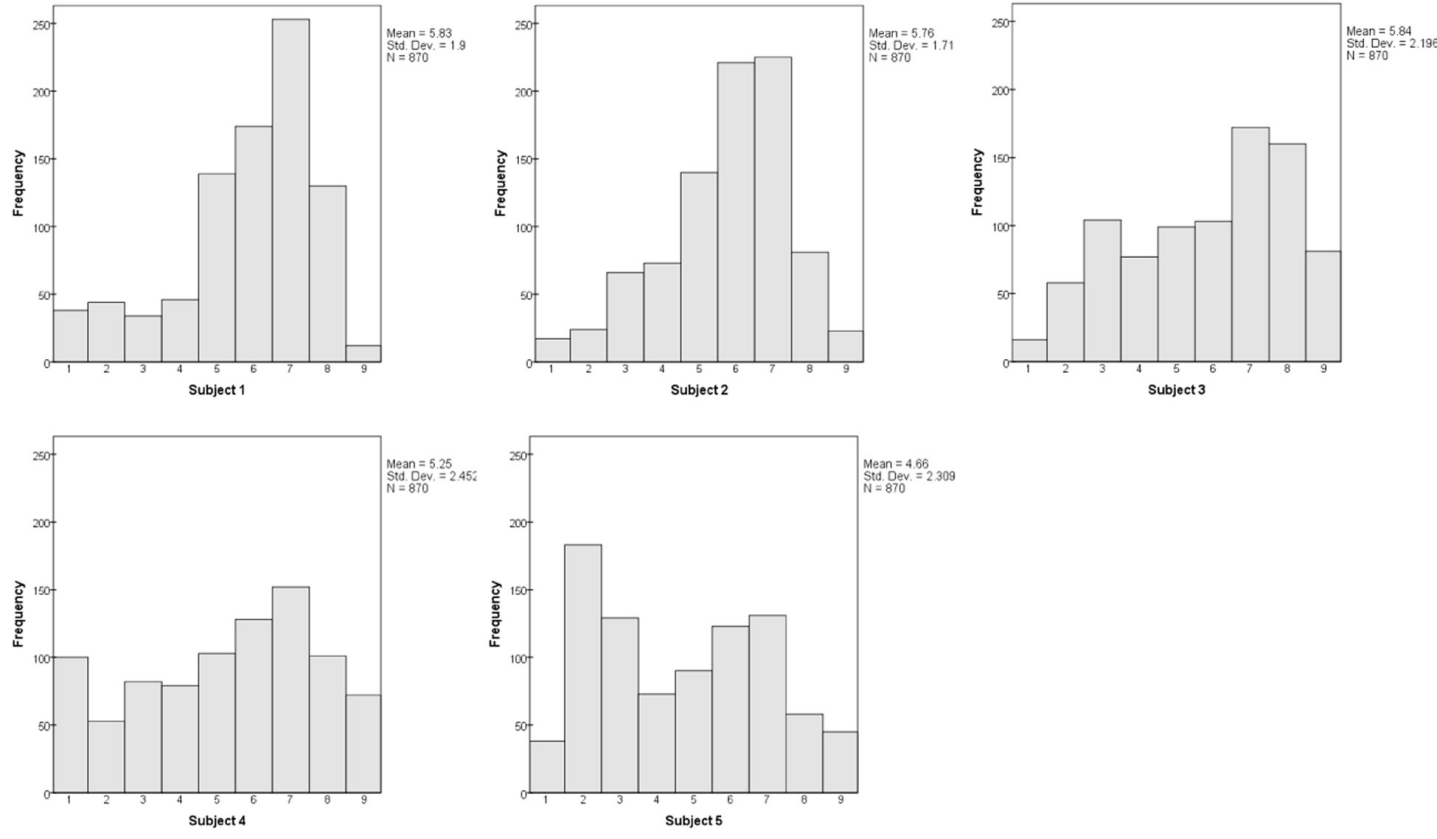
**Distributions of dissimilarity judgments from each subject**.

The individual distributions of dissimilarity ratings tend to be double-peaked (Figure 7), with the dominant peak containing the 600 bimodal pairs, while 270 *color-only* and *orientation-only* pairs form a smaller bulge of lower values. The distinctness of the second peak relies upon the *color-only* and *orientation-only* pairs having comparable dissimilarities and overlapping distributions; it is thus least distinct for Subject #2.

### Estimating minkowski parameter *p*

For each subject in turn, and for a given pair of colors (*a*:*b*), we obtain a mean dissimilarity Δ^*ab*^ by averaging the mean dissimilarity over the six appropriate *color-only* pairs (S^*a*^_*m*_: S^*b*^_*m*_) with 1 ≤ *m* ≤ 6, and over (S^*b*^_*m*_: S^*a*^_*m*_), i.e., the 12 presentations of that color combination as same-orientation bars (including the two presentations of a pair, left-right and right-left). By the same token, we obtain a mean dissimilarity Δ_*mn*_ for each pair of orientations by averaging the (5 × 2) combinations of that orientation pair as same-color bars (S^*a*^_*m*_: S^*a*^_*n*_) and (S^*a*^_*n*_: S^*a*^_*m*_) where 1 ≤ a ≤ 5. Inserting these values into Equation 1, with a given *p*, provides predicted dissimilarities for the bimodal stimulus pairs. We vary *p* and compare the predictions against the reported values. Note that this comparison relies on the raw data and does *not* involve the inter-point distances obtained in the MDS analysis.

**Figure 8:**
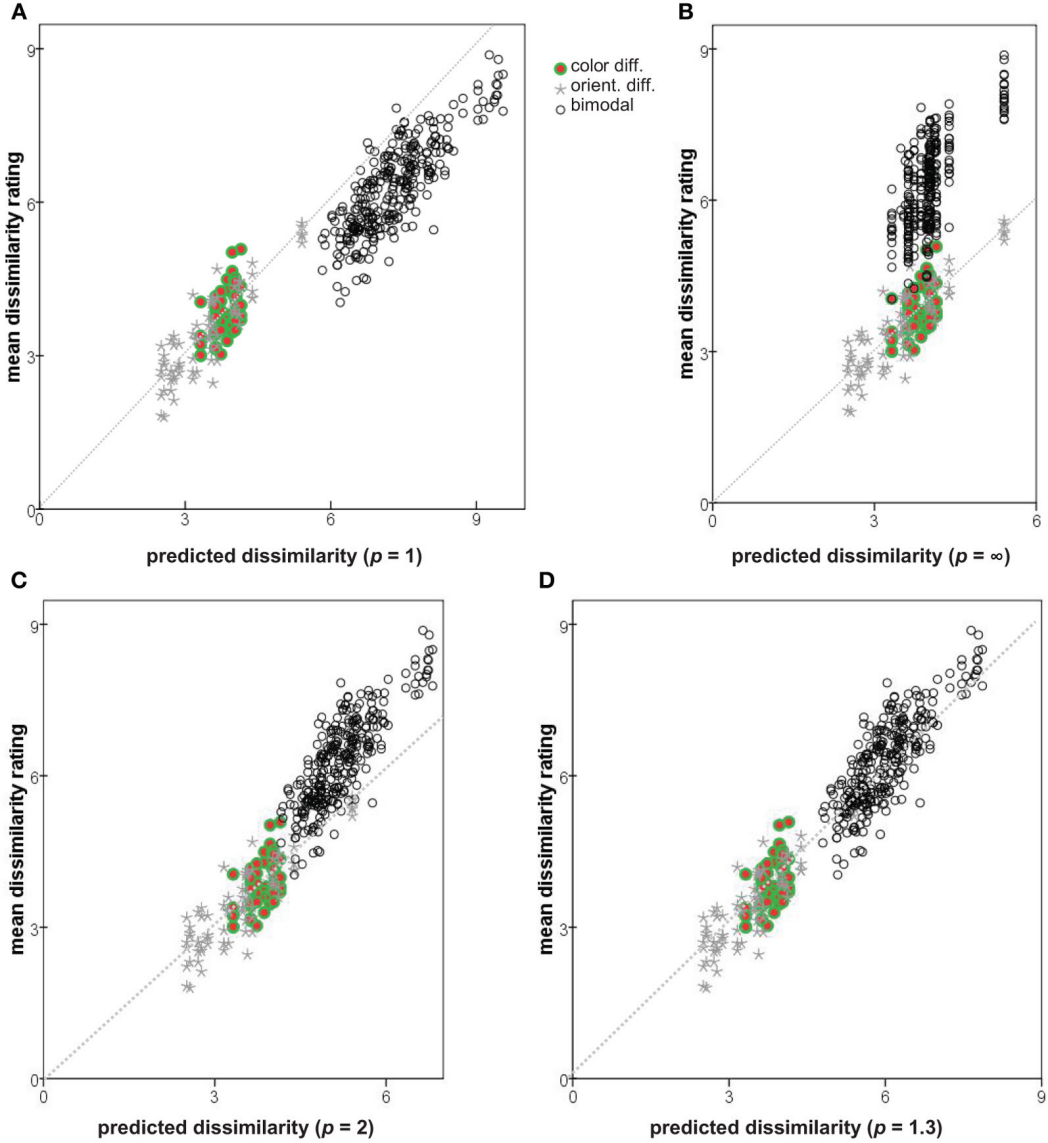
**Observed dissimilarity ratings plotted against values predicted by combining Δ_*mn*_ and Δ^*ab*^ with different Minkowski exponents. (A)** *p* = 1; **(B)** *p* → ∞; **(C)** *p* = 2; **(D)** *p* = 1.3. Diagonals shown as dotted lines.

Plotting the observed dissimilarity ratings (averaged over subjects) against the values predicted from Δ^*ab*^ and Δ_*mn*_ for three Minkowski metrics (*p* = 1, *p* → ∞, and *p* = 2) results in Figures 8A–C. Seemingly neither of the extreme metrics is ideal: the predicted dissimilarities for bimodal pairs are too large in (*p* = 1; Figure 8A) or too small (*p* → ∞; Figure 8B), in both cases introducing a discontinuity into the plot. The Euclidean metric (*p* = 2; Figure 8C) provides a better solution.

**Figure 9:**
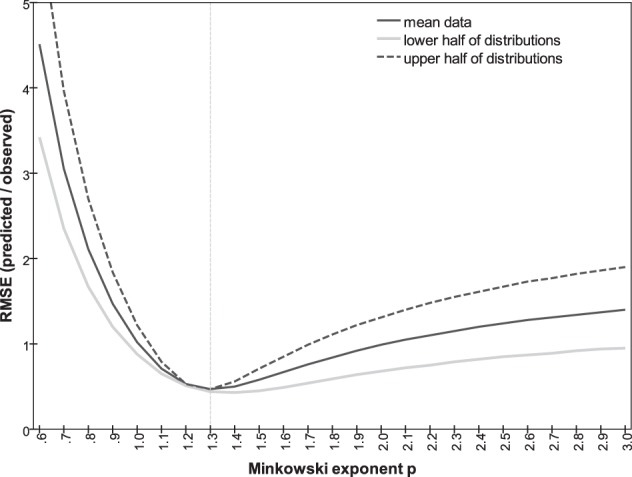
**RMSE between predicted and observed dissimilarities as a function of Minkowski exponent *p*, for all observations (solid line), for subset of smaller observations predicted from Δ_*mn*_ and Δ^*ab*^ less than mean value (gray line); for subset of larger observations predicted from Δ_*mn*_ and Δ^*ab*^ greater than mean value (dashed line)**.

In addition to the three Minkowski metrics named above, we explored the predictive power of intermediate metrics, varying exponent *p* between 0.7 and 3.0. Following Soto and Wasserman (2010) we use the root-mean square error (RMSE) to compare the predicted and actual observed dissimilarities, measuring the discontinuity in the predictions and how well they account for the observations. The RMSE as a function of *p* is plotted in Figure 9. A minimum of 0.47 is achieved at *p* = 1.30, compared to the values of 1.02 and 0.99 at *p* = 1 and 2, respectively. Note that RMSE is closely related to the summed residuals used by To et al. (2008, 2011) and the Pearson correlation *r* used by Shepard and Cermak (1973) (see also Dunn, 1983). Predicted dissimilarities for *p* = 1.3 are plotted against observations in Figure 8D.

An assumption in the argument is that the data are ratio-level, i.e., that each numerical rating is *proportional* to the perception of that dissimilarity. This is crucial for applying Equation 1 to the mean *orientation-only* and *color-only* ratings. The assumption cannot be tested directly. If, however, the subjects' rating responses are a non-linear function of perceptions, then a different *p* should be optimal for predicting the larger dissimilarities of the bimodal pairs generated by *orientation-only* and *color-only* dissimilarities that are both in the upper half of their distributions (i.e., Δ_*mn*_ > 3.43, Δ^*ab*^ > 3.94), where the perception/response curve presumably differs in slope. The optimal *p* should be different again when we take *orientation-only* and *color-only* ratings that are both in the *lower* half of their distributions and use them to predict the correspondingly smaller bimodal dissimilarities. Figure 9 plots, as a function of *p*, the correlation between predicted and empirical dissimilarities for these two subsets. Clearly the same Minkowski metric of *p* ~ 1.3 generates the dissimilarities for both subsets. Estimates of *p* could also be distorted if the dissimilarity ratings were interval-level, linear but including a non-zero constant; this possibility is harder to exclude.

**Figure 10:**
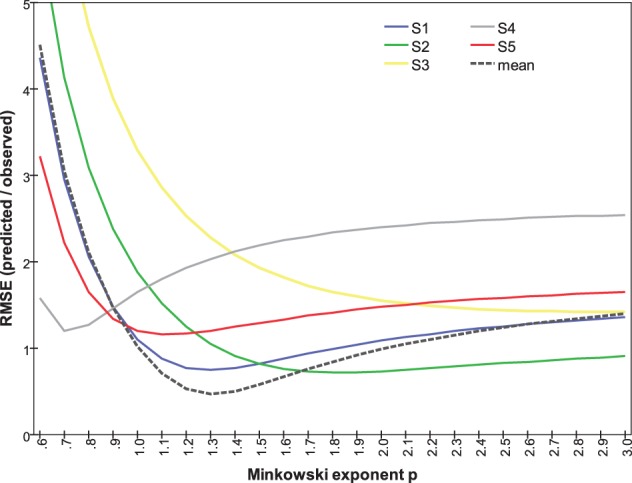
**RMSE between predicted and observed dissimilarities as a function of Minkowski exponent *p*, calculated separately for each subject's data**.

Repeating this analysis for individual subjects (Figure 10) shows substantial variation in the relationship between RMSE and *p*. It reveals, in particular that Subjects #1, #2, and #5 are governed by similar functions. Their optimal Minkowski exponents (1.3, 1.8, and 1.1, respectively; Table 3) correspond to a combination rule in which orientation and color are neither integral nor wholly separable. For Subject #4, with optimal *p* = 0.7, the function clearly indicates that orientation and color were separable, and indeed synergistic, so that the dissimilarity between stimuli differing on both attributes is greater than the sum of each attribute's dissimilarity in isolation. Finally, the optimal *p* = 3.1 for Subject #3 points to a combination rule that is closer to the Dominance metric.

## Discussion

On first glance the task of rating dissimilarities seems arbitrary and artificial. However, the relevance of the combination function that governs the underlying parameters is not limited to this task: integral and separable dimensions contribute in different ways to stimulus classification (Garner, 1974), classification errors (Shepard and Chang, 1963; Shepard, 1964), visual search (Treisman and Gormican, 1988), visual pop-out (Koene and Zhaoping, 2007), and signal detection (Ashby and Townsend, 1986). Moreover, perceptual dissimilarities bear upon the survival-centered problem of deciding whether or not the consequences of one stimulus generalize to a second. Shepard (1964, 1987) argued on *a priori* grounds that if an organism's perceptual process is attuned to regularities in its environment, it should follow either the *p* = 1 or *p* = 2 metric when it combines multiple sources of dissimilarity, depending on assumptions about selective attention and the consequential neighborhoods of the stimuli.

Hyman and Wells (1968) considered other conditions conducive to a low *p*. If the stimuli are processed as symbolic or verbal codes then the city-block metric would be the natural rule for obtaining their dissimilarities, with no interaction between the separately-encoded components of these descriptions (in addition, the discrete nature of the parameters of variation can be emphasized by spatial separation of the corresponding attributes). Indeed, the simple reductionist stimuli of the present study varied along orthogonal, “nameable” parameters of orientation and color. They lend themselves to a “verbal response strategy” where the representation of each stimulus is simplified by reducing it to higher-order symbolic labels (e.g., “60° + red”) and the parameters are processed as parallel verbal codes.

Tasks with a greater cognitive component can also shift integral dimensions to separable ones (Dunn, 1983; Foard and Nelson, 1984). Tversky and Gati (1982) went further, reporting a series of experiments where the dissimilarities could best be explained by a metric with *p* < 1, i.e., the attributes combined in a synergistic way (as with the present Subject #4).

Focusing like Shepard (1964) on cues and regularities in the visual environment, To et al. (2008, 2010) arrived at a different conclusion about dimensional integrality: the authors argue that changes in real-world scenes tend to be correlated (i.e., if one attribute of the scene has changed, it is likely that other attributes have changed also). Our perceptual mechanisms have the plasticity to recognize and exploit such correlations, creating the phenomenon of “cue recruitment” (Haijiang et al., 2006). The most efficient way of encoding such a change is the Dominance metric, in which the dissimilarity is determined by whichever attribute has changed most, suppressing other attributes since they provide little additional information. Indeed, dissimilarities between pictures of natural scenes were best fitted with Minkowski exponent *p* = 2.84 (To et al., 2008) or *p* = 2.48 (To et al., 2010), i.e., *p* > 2, indicating that an approximation to the Dominance metric was in place.

Further, a correlation between attributes is not the only condition that is conducive to a large value of *p*. Hyman and Wells (1967, 247) speculated that “speeding up the judgment process or otherwise overloading” the subject would increase *p* by causing competition and mutual masking among the dimensions. They wondered: “Does the apparent fit to the Euclidean metric in many judgment situations [i.e., *p* = 2 rather than *p* = 1 as might have been expected] indicate that [subject] is having trouble in extracting the information from both dimensions?” Complex differences in particular (as in To et al., 2008, 2010) might “saturate” the inter-stimulus dissimilarity. One complex scene manipulation—controlled by a single parameter, but changing multiple details of the scene—might leave the observer hard-pressed to attend to another simultaneous manipulation (thereby suppressing its contribution to the combined dissimilarity) simply by occupying the limited “bandwidth” of conscious comparisons. Foard and Nelson (1984) add stimulus duration and the task's nature to the factors affecting dimensional integrality.

We note in passing that the discriminative-limitation perspective predicts that *p* can be scale-dependent. Shepard's view (1987, Figure 4) of consequential neighborhoods makes the same prediction. For small enough differences between stimuli (or between stimulus and background), there is a threshold of discrimination where the detection of any change is limited by the specific sensory channel on which the difference is greatest (To et al., 2011). The contribution from any sub-threshold differences coded on other channels is small (in the case of probabilistic detection models) or zero. That is, *p* is large, approximating the Dominance metric as an asymptote. Thus, the neural channels that underlie some sensory domain can often be resolved with stimuli at the discrimination threshold, even if they merge in an isotropic continuum of integral dimensions at supra-threshold dissimilarities.

A tempting approach to the question is to apply MDS repeatedly with different Minkowski exponents *p*, choosing the *p* that minimizes badness-of-fit *stress*_1_. However, a confounding factor in calculations of *stress*_1_ is that the constraints of geometrical embedding are imposed most stringently in Euclidean geometry (*p* = 2). This is why the algorithms function most smoothly in Euclidean space. As Arabie notes (1991), MDS for *p* = 1 and *p* → ∞ turns a single *d*-dimensional optimization into a series of *d* one-dimensional optimizations (requiring a combinatorial attack rather than a simple steepest-descent algorithm), the problem persisting in milder form for any *p*≠ 2. A related property of Minkowski metrics for *p*≠ 2 is that small changes in the relative weighing or salience of the dimensions can produce abrupt, discontinuous changes in similarity or preference ranking (Shepard, 1964). Recent algorithms using Bayesian Likelihood rather than *stress*_1_ may finesse this problem (Okada and Shigemasu, 2010), but it is not clear how they apply to a “hybrid” geometry such as the present situation, in which *p* governs the combination of orientation and color, two internally-Euclidean subspaces.

One possibility is that the perception of dissimilarity emerges at an early stage of visual processing, from a neural locus where the signals of color and orientation are first combined; before attributes are subjected to parallel processing along separate pathways, and eventually re-integrated (Cavina-Pratesi et al., 2010). “Bottom-up” models based on visual search data allow the combination of dissimilarity contributions to approximate the Dominance metric (Zhaoping and May, 2007), but do not *require* such behavior, for the models do not place tight bounds on *p* (see also Nothdurft, 2000). Koene and Zhaoping (2007) postulated a “saliency map” in primary visual cortex in which the contrast between some combination of features (e.g., color C_1_ + orientation O_1_) and a background combination (C_2_ + O_2_) follows the Dominance metric, modified by detectors tuned to color + orientation conjunctions. The greater the input from conjunction detectors (relative to single-feature detectors), the further the metric is shifted toward the city-block model. Lateral inhibition from task-irrelevant variations in the background pattern reduces the city-block contribution (Zhaoping and May, 2007) and allows the proposed saliency map to behave more in line with the Dominance metric. Lateral inhibition of this kind could be a factor in difference judgments of the complex natural scenes used by To et al. (2008, 2010).

## Conclusions

MDS of dissimilarity ratings confirmed the expectation that orientation and color can be represented as separate subspaces, with *color-only* and *orientation-only* mean dissimilarities Δ^*ab*^ and Δ_*mn*_. Following Shepard and Cermak (1973), we combined these to obtain *p* directly (Figures 9, 10). The range of inter-individual variation of optimal exponents is substantial—between 0.7 and 3.1 (Table 3)—but comparable to ranges found in previous studies (cf. Dunn, 1983; Soto and Wasserman, 2010). Notably, the exponent is *p* < 2 for four of our five subjects, and for data averaged across subjects, so the orientation and color attributes had not become “integral,” nor merged their separate natures within an isotropic continuum. These values also conflict, to an even greater degree, with the results of To et al. (2008, 2010) from more complex scenes and manipulations.

The same conclusion—that color and orientation are not integral—emerges from the individual variations found by MDS. Specifically, the weight placed on color as a contribution to dissimilarity *varies* across subjects relative to the contribution from orientation (Table 3), with corresponding variations in the magnitudes of Δ^*ab*^ and Δ_*mn*_. There is no obvious relationship between these dimensional-salience parameters and the exponents *p*, nor is one to be expected. We note that for Subject #3, whose *p* > 2, the data showed lowest internal consistency (Table 2) and least compatibility with a geometrical model, i.e., highest *stress*_1_ (Table 3).

The obtained values also rule out the possibility that dissimilarities for these stimuli were determined *purely* by high-level, top-down cognitive operations, since the top-down symbolic-label model predicts *p* = 1, i.e., an absence of non-additive interactions between the two attributes. In practice the contribution of each attribute to total dissimilarity is affected by the value of the other attribute. If, for instance, a stimulus pair is separated by a smaller difference between their colors than between their orientations, then increasing the color difference will yield a relatively small increase in dissimilarity.

Possible artifacts were mentioned above that could increase *p* by encouraging mutual “masking” among the dimensions of variation. Of them, only the short time for responses applies (cf. Foard and Nelson, 1984): a change along either dimension is unlikely to saturate the capacity of visual processing, nor is there a background of task-irrelevant variations to inhibit the signal from feature-conjunction detectors in V1. Thus, it is unlikely that the subjects' actual values of *p* were much *lower* than these observed values.

It follows that the present results are not restricted to situations where the inter-stimulus variations involve clear-cut attributes, and a cognitive verbal-response strategy. We note also that Minkowski exponents *p* near to 1 have been reported even when the underlying parameters generating the stimuli are “relatively novel and difficult to verbalize—at least in any way that is general enough to extend beyond the immediate neighborhood of any one form” (Shepard and Cermak, 1973, 353).

The range of *p*-values across subjects is an interesting phenomenon in its own right, although it is an obstacle to drawing general, universally-applicable conclusions. One possible explanation is that a subject has access to several parallel strategies or processes, each comparing stimuli within a different Minkowski metric, with the judgment of dissimilarity being a combination of their outputs. Then the variations among subjects spring from weighting these outputs in different ratios. A possible role of top-down modulation in this weighting could be tested by manipulating the experimental instructions.

As noted earlier, Izmailov and Edrenkin (2010) reported dissimilarity data for 25 bar stimuli with five levels of orientation (0°, 30°, 60°, 90°, 120°) and of luminance (1, 2, 8, 32, and 64 cd/m^2^). We applied our analysis to their 50 orientation-only and 50 luminance-only pairs to predict the dissimilarities of bimodal pairs. The predictions were most accurate for *p* ~ 1.9. That is, in comparison to the present study, orientation and *luminance* appeared close to being integral. The departure demonstrates that there is nothing about the present approach that *forces* *p* < 2 as an outcome. Without further investigation, the reason for the different behavior of luminance is not obvious.

### Conflict of interest statement

The authors declare that the research was conducted in the absence of any commercial or financial relationships that could be construed as a potential conflict of interest.
